# Measuring Dyadic Interactions and Dining Environment in Dementia Mealtime Care: A Systematic Review of Instruments

**DOI:** 10.1093/geroni/igab046.2034

**Published:** 2021-12-17

**Authors:** Wen Liu, Sohyun Kim

**Affiliations:** 1 The University of Iowa College of Nursing, Iowa City, Iowa, United States; 2 University of Iowa, Iowa City, Iowa, United States

## Abstract

It is critical to use validated instruments to assess mealtime dyadic interactions and dining environment for people with dementia to evaluate the process and efficacy of mealtime interventions. However, the quantity and psychometric quality of such instruments are unknown. This systematic review aimed to synthesize the quantity and quality of instruments that assess dyadic interactions, physical environment, and/or social environment during dementia mealtime care. We searched Pubmed, CINAHL, AgeLine, PsychINFO, and Cochrane Library for records published between 1980-2020. Records were eligible if they included any instrument assessing concepts of interest (i.e., mealtime dyadic interactions, physical and/or social dining environment). From eligible records, eligible instruments originally developed or later modified to measure concepts of interest were identified, and instruments’ characteristics were extracted: 1) development process, 2) concept/construct assessed, 3) sample/setting, 4) administration method, 5) item description, 6) scoring format/interpretation, 7) reliability, and 8) validity. A newly developed tool was used to evaluate instruments’ psychometric quality. In total, 26 eligible instruments were identified. Seventeen instruments assessed dyadic interactions, 1 assessed only physical environment, and 8 assessed physical & social environment. All instruments were observational tools and scored as having low psychometric quality. Reasons for low psychometric quality included use of small sample size compared to the number of items, limited psychometric testing, and inadequate estimates. A number of instruments were developed and/or used to assess dyadic interactions, physical and/or social environment in dementia mealtime care. All instruments warrant further testing to accumulate psychometric evidence in larger diverse samples in different care settings.

